# 1-year results of lumbar spinal stenosis surgery in Finland: a national FinSpine register study

**DOI:** 10.2340/17453674.2025.42849

**Published:** 2025-02-14

**Authors:** Juho HATAKKA, Inari LAAKSONEN, Joel KOSTENSALO, Keijo T MÄKELÄ, Henri SALO, Katri PERNAA

**Affiliations:** 1 Department of Orthopaedics and Traumatology, Turku University Hospital, and University of Turku, Turku; 2 Natural Resources Institute Finland, Natural Resources, Joensuu; 3 Knowledge Brokers Department, Finnish Institute for Health and Welfare, Helsinki, Finland

## Abstract

**Background and purpose:**

While the rates of lumbar spinal stenosis (LSS) surgery have increased continuously internationally, the role of fusion surgery in the treatment of LSS has been under debate. We aimed to assess the outcome of LSS surgery at 1 year postoperatively and to compare decompression surgery with or without fusion based on the Finnish national spine register FinSpine data.

**Methods:**

FinSpine data of surgically treated LSS from 2015 to 2022 was included. The primary outcome was Oswestry Disability Index (ODI), and secondary ones were Visual Analogue Scale for leg and back pain. Predetermined minimal clinically important difference (MCID) for all outcome measures was used to assess the clinical significance of differences in outcomes. Propensity score matching was utilized to ensure that the treatment groups were comparable.

**Results:**

There were 8,647 LSS patients in the data, of whom 6,751 (77%) were the subject of decompression surgery. Over 90% of patients without spondylolisthesis received decompression alone. At 1-year follow-up, ODI was on average 20.6 (95% confidence interval [CI] 19.3–21.9]) for the fusion group and 23.3 (CI 22.5–24.0) for the decompression group. Differences in ODI, VAS leg pain, or VAS back pain were below the MCID. The share of patients reaching ODI percentage change score ≥ 30% was 74% (CI 71–78) in the fusion group and 66% (CI 63–68) in the decompression group.

**Conclusion:**

Most of the LSS patients experienced significant improvement after LSS surgery. We found no clinical differences between decompression surgery with and without fusion.

Lumbar spinal stenosis (LSS) is one of the most common indications for lumbar spine surgery. Degenerative narrowing of the lumbar spinal canal around the lumbar nerve roots, known as LSS, typically causes pain in the lower extremities, numbness and discomfort while standing or walking, and back pain. Over time the symptoms and disability of LSS patients remains mostly unchanged [1]. While some patients improve with non-surgical treatment, surgical decompression of neural structures has been shown to be an effective treatment for patients’ symptoms and disability [2,3].

Surgical quality registries can provide valuable information for assessing appropriateness of care, e.g., national spine registers of Sweden (Swespine), Norway (NORspine), Denmark (DaneSpine), and international Spine Tango by Eurospine. Especially in spine surgery, practice variations, improvement in quality of life after surgery, and the role of fusion surgery in LSS have been studied previously [4-6]. Some of these results indicating no benefit with additional fusion in decompressive surgery for LSS [4,7] may have led to the observed decrease in fusion rates in lumbar spine surgery in Finland since 2016 [8]. The Finnish national spine register FinSpine [9] enables researchers to access nationwide surgical data including PROMs of spine patients for the first time in Finland, making it possible to assess treatment and patient care of LSS as performed in everyday practice, to improve decision-making, compare results internationally, and to strengthen scientific evidence in this field of surgery.

The objective of this study was to study the outcome of LSS surgery in Finland at 1 year after the index operation and to compare the results of decompression surgery with or without concomitant fusion.

## Methods

### Study design

This study was based on the Finnish Spine Register (FinSpine) data and was reported according to the STROBE guidelines. Patients with a BMI over 60 were excluded, and for analysis of 1-year results of LSS surgery, only primary operations were allowed. All fusion techniques were included, and patients were not pooled by either decompression or fusion levels.

### Setting, data sources, and participants

FinSpine was initiated in 2016, and the register has been funded by the Finnish Institute of Health and Welfare from the beginning of 2023. FinSpine data from the year 2015 to 2022 was included, thus including the register pilot year 2015. The precise description of FinSpine data content, data collection, and coverage has been described previously by Marjamaa et al. [9]. All the primary operations with lumbar spine as the operation target and LSS as the operative diagnosis were included. The LSS diagnosis is divided into central canal stenosis and lateral recess stenosis in the register, and these 2 entities are further divided into < or > 3 mm spondylolisthesis. All patients were included in the study. In the presence of other diagnoses, such as tumors, trauma, infections, and any type of spinal deformity, the patients’ records were excluded. FinSpine data has been validated against, forth and back, hospital discharge register (HILMO) and proved to be more accurate than HILMO [9]; intra- and interobserver validation has started.

The registry data included most of the spine surgeries carried out in Finland, especially after the first years of the registry being online. The coverage was 83% in public hospitals, the completeness was 86%, and 90% of the surgeries in public hospitals as of 2022 and the compliance of surgeons has been 80% [9].

Patient-specific data included age, sex, body mass index (BMI), usage of nicotine products, duration of pain, usage of pain medication, and employment status.

### Outcomes

To assess the outcome of LSS surgery at 1 year, we used Oswestry Disability Index (ODI, 0–100 worst) as the primary outcome measure: the ODI is used for measuring disability and quality of life in subjects with low back pain and is recommended by ICHOM [10]. A Visual Analogue Scale (VAS, 0–100 worst) for back pain and leg pain was used for the secondary outcome measures. Patient satisfaction was assessed by 3 questions: “Would you undergo surgery again? ,” “Overall satisfaction,” and “Are your present symptoms better, worse, or the same?”

Baseline data on patients, PROMs, and patient characteristics were collected 0–2 months prior to operation and follow-up data at 3 months and 1 year after operation.

### Variables

The minimal clinically important difference (MCID) describes the smallest change in a PROM score, which is meaningful for an individual patient. Various MCID values from 6 to 15 have been reported for ODI, depending on method of determination and population [11-13] with a tendency for smaller values. MCID of VAS for lower back pain has been reported to be 15; the same was used for VAS leg pain [14]. ODI is the primary outcome measure, followed by the VAS measures, with other measures being secondary.

Percentage change score of an outcome measure has the advantage of taking into account the baseline score of the measurement tool and combined with acceptable symptom state has been shown to reflect clinically important outcomes better than the score change itself [15]. For ODI, a significant percentage change score has been determined to be ≥ 30% [14,15]. The acceptable symptom state at 1-year follow-up for ODI varies depending on the current condition, but for spinal stenosis and degenerative spondylolisthesis it has been set at ≤ 24 [15,16].

### Statistics

Because of large sample size, over 8,000 patients from which 604 matched pairs (n = 1,208) with full data were formed, statistically significant differences between the outcomes will be present, which makes clinically significant assessment relevant. The statistical power for MCID differences was estimated to be > 95% for both ODI and the VAS measures based on 10,000 simulated datasets.

As this was not a randomized controlled trial, the patient demographics differed between the decompression and the fusion group. To control for these, we created a dataset where each fusion patient with full ODI information (n = 604) was matched with a decompression patient using propensity score matching. The matching was done using the MatchIt package (https://kosukeimai.github.io/MatchIt/index.html), with age, sex, preoperative ODI score, and type of LSS (recessive or central) as covariates to be balanced. The results of the matching are shown in Supplementary data (Figure S1). Other potentially helpful covariates such as BMI, smoking, and use of pain medication had a significant amount of missing data and were thus not used. Differences between groups for dichotomous variables were tested using Fisher’s exact test. Because the distributions of the primary outcome variables deviated significantly from normality, differences in group means, their 95% confidence intervals (CI), and P values were estimated using pairwise (i.e., sampling by matched pairs) non-parametric bootstrap. The interpretation of clinical significance of a difference was predetermined prior to the analyses so that for a difference to be clinically significant, the 95% CI for the mean difference should be fully over the limit for MCID. If the 95% CI was fully below MCID, this was taken as evidence that the average difference is unlikely to be clinically significant. If the 95% CI included the MCID value, the evidence would be insufficient to rule either way. All statistical analyses were carried out using the statistical software R version 4.3.2 (R Foundation for Statistical Computing, Vienna, Austria).

### Use of AI, funding, and disclosures

Ai tools were not used. JH and IL have received funding from State Research Funding of South-western Finland; IL received support from Arthrex Finland Ltd for a scientific meeting. KP is a member of the FinSpine Steering Group. The authors have no conflict of interest to declare. Complete disclosure of interest forms according to ICMJE are available on the article page, doi: 10.2340/17453674.2025.42849

## Results

**Figure 1:**
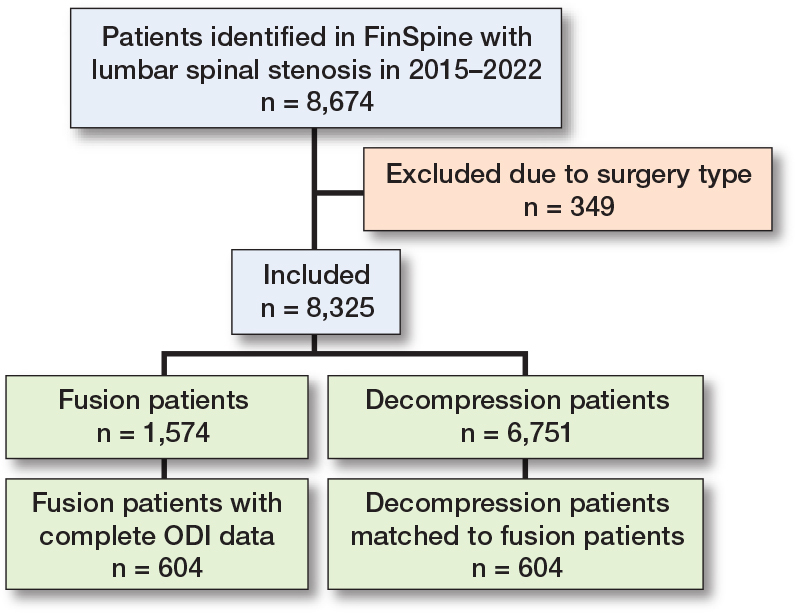
Flowchart of patients included in the study. Complete ODI data refers to patients who have ODI measurements both preoperatively and 1-year postoperatively. The matched decompression patients have full ODI data and have been matched with 1:1 propensity-score matching.

A flowchart representing the inclusion and exclusion of patients in the study is given in Figure 1. There were 8,674 patients with an LSS diagnosis in the data. Decompression procedure was undertaken for 6,751 patients (77%) and decompression with concomitant fusion for 1,574 patients (18%). The remaining 349 patients (4%) were subjects of another type of surgery, or their surgical data was missing (Table 1).

**Table 1:** Demographics of all lumbar spinal stenosis patients in the FinSpine registry, as well as patients treated with decompression, and patients treated with decompression and fusion (Decom. + fusion). The Decom. (matched) column refers to the subset of decompression patients, which was matched to the fusion group using 1:1 propensity-score matching. Values are count (%) unless otherwise specified

Variable	All	Decom.	Decom. + fusion	Decom. (matched)	Decom. + fusion (full ODI)
n	8,674	6,751 (77)	1,574 (18)	604 (7.0)	604 (7.0)
Age, mean (SD)	68 (11)	68 (11)	66 (10)	64 (11)	64 (10)
Female	4,955 (57)	3,520 (52)	1226 (78)	463 (77)	466 (77)
BMI (SD)	28.4 (4.6)	28.5 (4.6)	28.2 (4.5)	28.9 (4.8)	28.1 (4.6)
Nicotine	667 (15)	529 (15)	114 (12)	76 (13)	78 (14)
Pain duration					
< 6 weeks	112 (2.5)	101 (3.0)	8 (0.9)	16 (2.7)	6 (1.0)
6–12 weeks	198 (4.5)	168 (5.0)	23 (2.6)	27 (4.6)	13 (2.3)
3–12 months	1,237 (28)	997 (30)	204 (23)	183 (32)	128 (23)
>12 months	2,846 (65)	2,102 (62)	660 (74)	354 (61)	415 (74)
Usage of pain medication					
No/occasional	1,674 (38)	1,344 (40)	278 (31)	197 (34)	183 (33)
Regular	2,726 (62)	2,030 (60)	618 (69)	380 (66)	379 (67)
Employment status					
Working	1,035 (23)	760 (22)	240 (26)	171 (29)	150 (26)
Unable to work	460 (10)	317 (9.2)	124 (14)	83 (14)	84 (15)
Unemployed	148 (3.3)	110 (3.2)	36 (3.9)	18 (3.1)	24 (4.2)
Retired	2,836 (63)	2,247 (65)	514 (56)	313 (53)	315 (55)
LSS definition (spondylolisthesis)					
Central canal					
< 3 mm	4,403 (51)	4,023 (60)	202 (13)	322 (53)	75 (12)
> 3 mm	2,137 (25)	1,042 (15)	1,020 (65)	121 (20)	379 (63)
Lateral recess					
< 3 mm	1,579 (18)	1,452 (22)	55 (3.5)	135 (22)	28 (4.6)
> 3 mm	556 (6.4)	234 (3.5)	297 (19)	26 (4.3)	122 (20)
Other diagnoses					
Disc degeneration	91 (1.0)	46 (0.7)	37 (2.4)	4 (0.7)	17 (2.8)
Disc herniation	697 (8.0)	603 (8.9)	52 (3.3)	54 (8.9)	23 (3.8)
Other	1,817 (21)	1,234 (18)	471 (30)	114 (19)	200 (33)

The proportion of female subjects 1,226 (78%) was larger in the decompression with fusion group compared with the decompression group 3,520 (52%). Furthermore, patients’ symptom duration had been longer, and they had had more frequent need of pain medication in the decompression with fusion group. There were no significant differences in BMI and usage of nicotine products between the 2 groups. Length of inpatient care in days (SD) was 3.4 (2.0) in the decompression group and 4.3 (2.0) in the decompression and fusion group.

### Descriptive data

Central canal stenosis was the most common diagnosis, 51% among all patients (n = 4,403), followed by central canal stenosis with concomitant spondylolisthesis at 25% (n = 2,137), lateral recess stenosis at 18% (n = 1,579) and lateral recess stenosis with spondylolisthesis at 6% (n = 556). Patients with central or lateral recess stenosis without spondylolisthesis received decompression in 4,023 (91%) and 1,452 (92%) of the cases, respectively. For patients with central canal stenosis with spondylolisthesis, decompression and decompression with fusion were equally common procedures. Patients with lateral recess stenosis with spondylolisthesis were prone to receive decompression with fusion: 297 out of 556 patients (53%).

### Outcome data

Baseline response rate for ODI was 54% (n = 4,663), for VAS leg pain 50% (n = 4,307), and for VAS back pain 50% (n = 4,305). Response rates at 1-year follow-up were 38% (n = 3,301), 34% (n = 2,926), and 35% (n = 3,003), respectively.

Preoperative mean ODI was 44.0 (CI 42.9–45.0) in the fusion group compared with 42.2 (CI 41.7–42.8) in the decompression group, and thus there was a 1.8-point difference between the groups. When looking at the fusion patients who had answered the ODI preoperatively and at the 1-year mark, the preoperative average was 43.5 (CI 42.2–44.8) and for the decompression patients matched using propensity score matching 42.9 (CI 41.6–44.1), i.e., the difference in preoperative ODI is on average 0.6 points.

### Primary outcome

**Figure 2:**
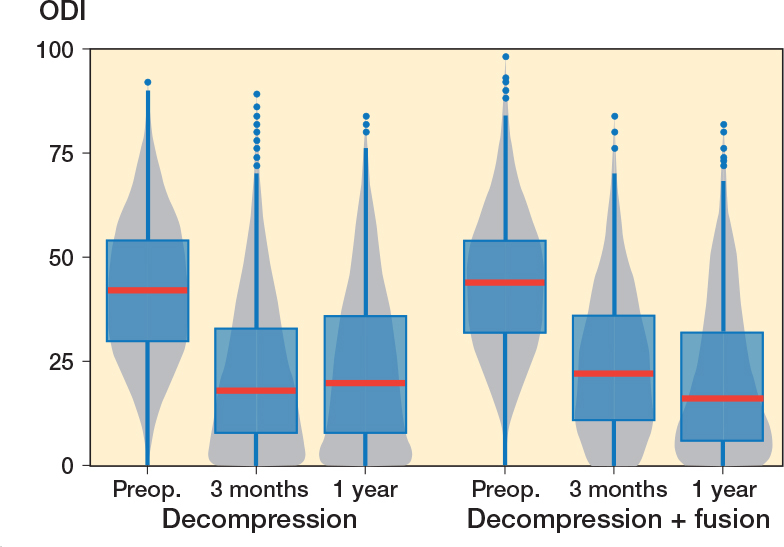
Patient-reported Oswestry Disability Index (ODI) values for lumbar spinal stenosis patients in the FinSpine registry treated with decompression, and decompression with fusion. The horizontal line corresponds to the median, the box to the lower and upper quartiles and the whiskers correspond to the minimum and maximum. Outliers (i.e., observations over 1.5 times the interquartile range from the upper quartile) are shown as dots. The gray zone is the corresponding violin plot showing the distribution of observations, with wider gray zones representing a larger number of observations and narrow parts few observations.

The functional outcome at 1-year follow-up for ODI was 20.6 (CI 19.3–21.9) for the fusion group and 23.3 (CI 22.5–24.0) for the decompression group (Figure 2). The share of patients reaching a percentage change score greater or equal to 30% was 67% in the whole group, 74% in the fusion group, and 66% in the decompression group. A follow-up score of less or equal to 24 was reached by 60%, 66%, and 59% of patients, respectively (Table 2). For the propensity-score matched data, the decompression group did slightly worse, with ODI score improvement being on average 2.4 (CI 0.5–4.4) less than for the fusion group.

**Table 2:** Surgical outcomes by PROM and treatment group at 1 year. The P values are from Fisher’s exact test (F) or non-parametric bootstrap (B) depending on whether the outcome measure was dichotomous or not

Item	All	Decom.	Decom. + fusion	Decom. (matched)	Decom. + fusion (full ODI)	Difference Decom. – decom. + fusion	P value
ODI							
Answered (%)							
Preoperatively	54	53	60	100	100		
At 1-year F-U	38	36	48	100	100		
Preoperative, mean (SD)	42.6 (16.5)	42.2 (16.5)	44.0 (16.4)	42.9 (15.9)	43.5 (16.3)	–0.7 (22.8)	0.5 **^B^**
[CI]	[42.2 to 43.1]	[41.7 to 42.8]	[42.9 to 45.0]	[41.6 to 44.1]	[42.2 to 44.8]	[–2.5 to 1.1]	
3-month, mean (SD)	22.6 (17.7)	21.8 (17.9)	24.5 (16.7)	21.9 (17.1)	25.2 (17.1)	–3.3 (25.2)	0.03 **^B^**
[CI]	[22.0 to 23.2]	[21.2 to 22.5]	[23.4 to 25.6]	[20.2 to 23.6]	[23.6 to 26.8]	[–5.7 to –0.9]	
1-year, mean (SD)	22.8 (18.6)	23.3 (18.6)	20.6 (17.8)	22.5 (17.8)	20.7 (17.8)	1.8 (25.8)	0.09 **^B^**
[CI]	[22.1 to 23.4]	[22.5 to 24.0]	[19.3 to 21.9]	[21.1 to 23.9]	[19.3 to 22.1]	[–0.3 to 3.8]	
1-year value – preoperative value (difference)							
mean (SD)	–19.5 (18.3)	–18.5 (18.3)	–22.8 (17.9)	–20.4 (18.0)	–22.8 (17.9)	2.4 (24.9)	0.02 **^B^**
[CI]	[–20.2 to –18.8]	[–19.4 to –17.7]	[–24.2 to –21.4]	[–21.8 to –18.9]	[–24.2 to –21.4]	[0.5 to 4.4]	
1-year change > 15 **^a^** (%)	58	55	69	59	69	–10 [–15 to –4]	<0.001 **^F^**
1-year change ≥ 30% **^b^** (%)	67	66	74	68	74	–6 [–11 to –1]	0.02 **^F^**
1-year value ≤ 24 **^c^** (%)	60	59	66	60	66	–6 [–11 to 0]	0.05 **^F^**
VAS leg pain							
Answered (%)							
Preoperatively	50	48	57	89	94		
At 1–year F-U	34	32	42	85	88		
Preoperative, mean (SD)	62.5 (26.9)	62.4 (26.9)	62.7 (27.0)	61.1 (27.7)	60.9 (27.7)	0.2 (40.0)	0.9 **^B^**
[CI]	[61.7 to 63.3]	[61.5 to 63.3]	[60.9 to 64.4]	[58.6 to 63.2]	[58.6 to 63.2]	[–3.2 to 3.4]	
3-month, mean (SD)	29.8 (29.1)	31.1 (29.6)	25.5 (26.9)	31.8 (29.5)	24.6 (26.9)	7.2 (40.9)	<0.001 **^B^**
[CI]	[28.8 to 30.8]	[29.8 to 32.3]	[23.6 to 27.5]	[28.6 to 34.9]	[21.8 to 27.3]	[3.1 to 11.3]	
1-year, mean (SD)	35.5 (30.8)	36.5 (30.9)	31.6 (29.5)	37.2 (31.0)	31.8 (29.4)	5.4 (41.8)	0.006 **^B^**
[CI]	[34.4 to 36.6]	[35.2 to 37.8]	[29.4 to 33.9]	[34.5 to 39.8]	[29.3 to 34.3]	[1.7 to 8.9]	
1-year value – preoperative value (difference)							
mean (SD)	–25.4 (36.6)	–24.2 (36.6)	–29.5 (36.1)	–24.2 (36.0)	–29.1 (36.1)	4.9 (51.3)	0.04 **^B^**
[CI)	[–26.9 to 23.8]	[–26.1 to –22.4]	[–32.6 to –26.4]	[–27.5 to –20.9]	[32.3 to –26,0]	[0.3 to 9.5]	
1-year change > 15 **^a^** (%)	60	59	62	58	62	–3 [–10, to 3]	0.3 **^F^**
VAS back pain							
Answered (%)							
Preoperatively	50	48	57	91	93		
AT 1-year F-U	35	33	43	91	90		
Preoperative, mean (SD)	58.3 (27.3)	57.1 (27.6)	62.8 (25.7)	58.0 (27.8)	61.0 (25.9)	–3.1 (38.7)	0.06 **^B^**
[CI]	[57.5 to 59.1]	[56.2, 58.1]	[61.1 to 64.5]	[55.6 to 60.3]	[58.9, 63.2]	[–6.3 to 0.1]	
3-month, mean (SD)	26.6 (26.0)	27.3 (26.5)	24.1 (24.0)	27.0 (26.2)	23.5 (24.1)	3.6 (34.7)	0.05 **^B^**
[CI]	[25.7 to 27.5]	[26.2, 28.4]	[22.4 to 25.8]	[24.3 to 29.8]	[21.1, 25.9]	[0.0 to 7.0]	
1-year, mean (SD)	31.7 (28.8)	32.9 (29.2)	27.4 (27.0)	34.2 (29.0)	28.0 (27.2)	6.2 (41.0)	<0.001 **^B^**
[CI]	[30.6 to 32.7]	[31.7, 34.1]	[25.4 to 29.4]	[31.8 to 36.6]	[25.7, 30.3]	[2.8 to 9.6]	
1-year value – preoperative value (difference)							
mean (SD)	–25.0 (34.8)	–22.2 (34.7)	–33.6 (33.6)	–24.3 (34.6)	–33.3 (33.7)	9.0 (49.4)	<0.001 **^B^**
[CI]	[–26.5 to –23.5]	[–23.9, –20.5]	[–36.3 to –30.5]	[–27.3 to –21.3]	[–36.2 to –30.4]	[4.8, to 13.2]	
1-year change > 15 **^a^** (%)	59	57	68	58	68	–10 [–16 to –4]	0.001 **^F^**

Decom. = decompression; F-U = follow-up.

a Minimal clinically important difference (MCID).

b Significant percentage change score.

c Acceptable symptom state.

**Figure 3:**
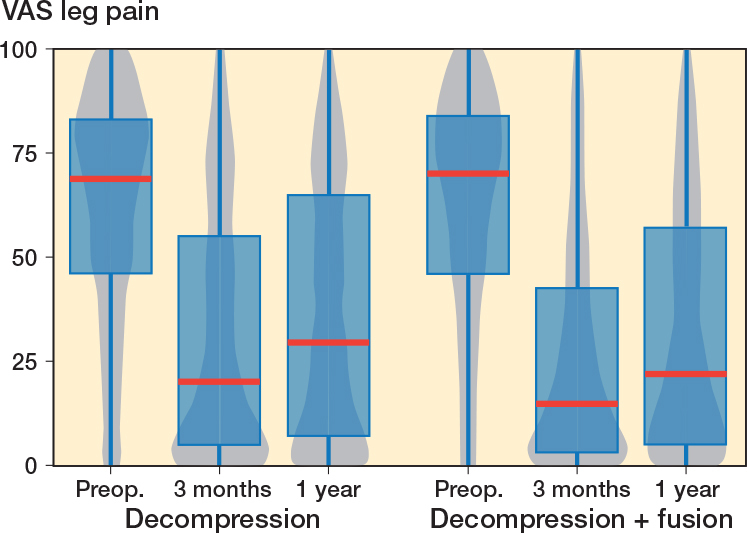
Patient-reported Visual Analogue Scale (VAS) values for leg pain for lumbar spinal stenosis patients in the FinSpine registry treated with decompression, and decompression with fusion. Details of the visual representation are given in the caption for Figure 2.

**Figure 4:**
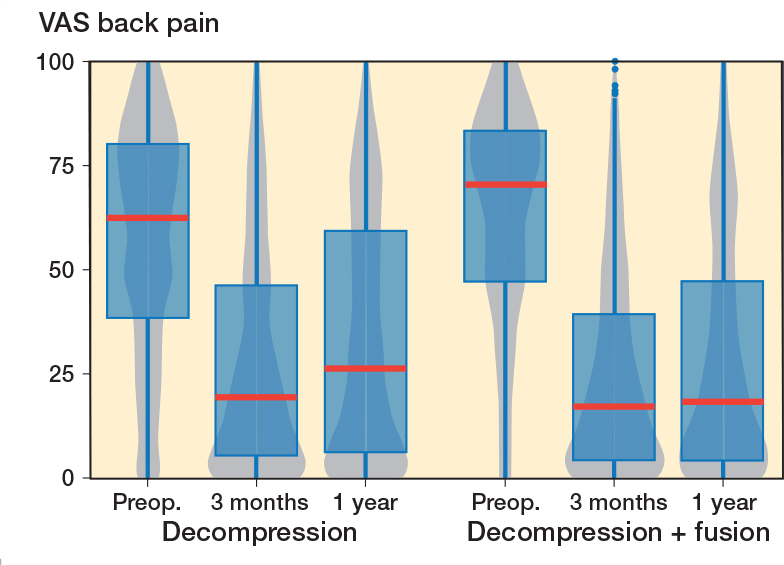
Patient-reported Visual Analogue Scale (VAS) values for lower back pain for lumbar spinal stenosis patients in the FinSpine-registry treated with decompression, and decompression and fusion (Deco. + fusion). Details of the visual representation are given in the caption of Figure 2.

In the fusion group, VAS leg pain mean improvement was 29.5 (CI 26.4–32.6) points (Figure 3) and for VAS back pain 33.6 (CI 30.5–36.3) points (Figure 4), while in the decompression group the corresponding improvements were 24.2 (CI 22.4–26.1) points and 22.2 (CI 20.5–23.9) respectively over the 1-year follow-up period. For the matched data the improvement in the decompression group compared with the fusion group was 4.9 (CI 0.3–9.5) for VAS leg pain and 9.0 (CI 4.8–13.2) for VAS back pain. However, it should be noticed that the fusion patient started with 3 points higher VAS back pain, while the difference was only 0.2 points for VAS leg pain. As a sensitivity analysis we ran the analysis so that instead of preoperative ODI we used preoperative VAS back pain as a covariate in the propensity score matching. In the resulting data set the VAS back pain was 1.4 points higher in the other direction, and the difference was mitigated to 6.2 (CI 2.2–10.1) in favor of the fusion.

The majority of patients were satisfied with their surgical outcome. Among all responders, 89% would undergo surgery again, 73% were satisfied, and 76% felt their back and/or leg symptoms were better than before surgery (Table 3). Satisfaction was a bit more prominent in the fusion group compared with the decompression group, 77% vs 71%, respectively.

**Table 3:** Overall satisfaction by treatment group at 1 year. Values are count (%)

Overall satisfaction at 1 year F-U	All	Decom.	Decom. + fusion	Decom. (matched)	Decom. + fusion (full ODI)
“Would you undergo surgery again?”					
Yes	2,555 (89)	1,920 (89)	559 (89)	486 (90)	456 (88)
No	64 (2.2)	48 (2.2)	15 (2.4)	7 (1.3)	12 (2.3)
Not sure	246 (8.6)	178 (8.3)	55 (8.7)	46 (8.5)	48 (9.3)
Satisfaction					
Satisfied	2,070 (73)	1,518 (71)	485 (77)	378 (71)	398 (77)
Unsatisfied	226 (7.9)	179 (8.4)	44 (6.8)	36 (6.8)	36 (6.9)
Not sure	553 (19)	433 (20)	101 (16)	118 (22)	84 (16)
Present back/leg symptoms					
Better	2,174 (76)	1,588 (74)	520 (83)	390 (73)	424 (82)
Worse	210 (7.4)	169 (7.9)	31 (4.9)	44 (8.3)	27 (5.2)
Same	473 (17)	382 (18)	78 (12)	98 (18)	67 (13)

For abbreviations, see Table 2.

### Other analyses

In the non-responder analysis those who did not answer at the 1-year follow up were older and more likely to use nicotine products than other responders. Their baseline ODI, VAS leg pain, and VAS back pain were slightly worse, but the differences were not clinically significant.

In subgroup analysis for central and recessive stenosis, ODI mean improvement was 20.2 (CI 18.6–21.9) in the central stenosis decompression group and 22.5 (CI 20.8–24.1) in the central stenosis with concomitant fusion group, while matched results in recessive stenosis were 20.7 (CI 17.9–23.6) and 23.9 (CI 21.2–26.6) respectively (Table S3, see Supplementary data).

## Discussion

We aimed to assess results of LSS surgery at 1 year postoperatively and to compare decompression surgery with or without fusion. We found that most of the LSS patients experienced significant improvement after LSS surgery but there were no clinical differences between decompression surgery with and without fusion.

Both treatment groups acquired improvement in ODI, VAS leg pain, and VAS back pain greater than MCID. Compared with decompression, fused patients managed equally in terms of ODI reduction.

Of all patients, 59% had a clinically significant reduction in back pain: 57% of patients in the decompression group and 68% of patients in the decompression and fusion group. The difference between the 2 surgical groups’ mean changes was 9.0, which is below the MCID of 15 for VAS, hence fusion cannot be regarded as improving back pain significantly better than decompression alone. Earlier, in a study by Srinivas et al., 68% of patients and in a study by Crawford III et al. 79.9 to 85.8% of patients remained clinically significantly improved in back pain over a year [17,18]. Although Srinivas et al. used NRS and its MCID of 2.0 points for their CSORN study, they found no clinically meaningful effect of additional fusion on back pain at 1 year after surgery, which is in agreement with our results (18). Improvement in leg pain was greater in the fusion group, but not statistically significant; neither was the difference in patients reaching clinically important change. Although clinical symptoms of central and recessive stenosis might represent difference, we found no difference in outcome in subgroup analysis at 1 year.

Over 90% of patients without spondylolisthesis, nearly half of the patients with central canal stenosis and spondylolisthesis, receive decompression alone, while decompression with fusion was the most common procedure for patients with lateral recess stenosis and spondylolisthesis. Almost 80% of fused patients were female and they had slightly higher baseline ODI (44.0 vs 42.2), their symptom duration had been longer, and they had more frequent use of pain medication.

In former register studies, fusion rates in LSS surgery have varied remarkably. In a comparative study of a Norwegian register and a clinical database from Boston, USA by Lønne et al. [19], the overall fusion rate was 13.9% in Norway and 51% in Boston. The fusion rate for patients with and without spondylolisthesis in our study was 49% and 5%, which are close to those in Norway at 47% and 5% respectively [20]. Lumbar fusion rates in Finland have declined from 2016 according to a study by Ponkilainen et al. [8], assumedly due to recent studies comparing fusion vs decompression and finding no evidence of superiority of fusion. Our data included surgeries mostly after this decline in fusion rates; thus, this might have influenced PROM results in our data as well.

The role of fusion in LSS surgery has been debated in past years. Recent RCTs on the effect of additional fusion in LSS surgery by Försth et al. [7] and Austevoll et al. [21] have not found fusion to be superior compared with decompression alone, while Ghogawala et al. [22] found fusion to result in slightly better outcomes in mild spondylolisthesis. Instability has been proposed to be an indication for fusion, but its definition varies greatly. In our study, there was greater reduction of back pain in the fusion group, which vanished in subgroup and sensitivity analysis, leaving no differences between groups despite spondylolisthesis. In future studies, closer evaluation of subgroups, to find possible explanations for the need for fusion, should be made.

### Limitations

Low response rate for PROMs at the 1-year follow-up is the main limitation in our study, and some precaution must be applied while assessing the results, especially in that self-selection bias might be present, and also when there was great variance in response rates between treatment groups, which may have influenced the results and limited the generalizability of the results. Van Hooff at al. proposed 60–80% response rate at 1-year follow-up to reduce bias in their study to enhance the reliability of evidence based on spinal surgery [6]. In 2022, the coverage was 86% of all spine surgeries performed in Finland, 90% of the surgeries in public hospitals, and the compliance of surgeons was 80% [9].

While we accounted for the matched design for continuous variables, such as ODI and VAS change, by using pairwise bootstrap sampling, the analysis for dichotomous variables did not account for the matched design. Furthermore, while the patients were well matched based on the covariates considered, it is possible that there could be some systematic differences in other variables not present in the data.

Non-responder analysis, conducted due to low baseline response rate, showed that our non-responders were slightly older than responders, which is the opposite of findings in previous studies by Lønne et al. and Solberg et al. [20,23]. This finding is probably insignificant but might reflect that our follow-up data is collected by electronic platform (link provided via SMS message).

### Conclusion

Most of the LSS patients experienced significant improvement after LSS surgery. We found no clinical differences between decompression surgery with and without fusion.

### Supplementary data

Tables S1–S5 and Figures S1–S6 are available as supplementary data on the article page including detailed information on non-responder analysis, propensity score matching and subgroup analysis, doi: 10.2340/17453674.2025.42849

## Supplementary Material



## Notes

JH, IL, HS, KTM, JK, and KP designed the protocol and methods. HS collected the data. JK performed the formal analysis and created the visualizations. All authors interpreted the results. JH, IL, JK, and KP wrote the manuscript. All authors contributed to the revision of the manuscript.

The authors thank the FinSpine steering group.

Handling co-editors: Paul Gerdhem and Philippe Wagner

*Acta* thanks Peter Försth, Konstantinos Pazarlis and Ane Simony for help with peer review of this manuscript.
