# Supplementary material for: 1-year results of lumbar spinal stenosis surgery in Finland: a national FinSpine register study
Source: Acta Orthop. 2025 Feb 14;96:154–60. doi: 10.2340/17453674.2025.42849 (PMC11829219; doi:10.2340/17453674.2025.42849)
Supplement: Supplementary file 1 [file ActaO-96-42849-s1.pdf]

AO-2024-67 - (17729) One-year results of lumbar spinal stenosis surgery in Finland a national FinSpine register study

SUPPLEMENTARY MATERIAL

Table S1. Demographic details between the patients who answered and did not answer the PROM questionnaire preoperatively. The P-values are from Fisher's exact test (F), non-parametric bootstrap (B), or a chi-squared test (C) depending on the distribution of the variable. Values are count (%) unless otherwise specified

Variable		Respondent	Non-respondent	P value
Total n, %		4,663 (54)	4,011 (46)	
Age, mean (SD)		66 (11)	70 (11)	<0.001 (B)
Female		2,653 (57)	2,302 (57)	0.7 (F)
BMI (SD)		28.4 (4.6)	28.8 (4.4)	0.3 (B)
Nicotine		640 (15)	27 (17) (n=163)	0.5 (F)
Pain duration	< 6 weeks	108 (2.3)	4 (5.1)	<0.001 (C)
	6–12 weeks	192 (4.1)	6 (7.7)	
	3–12 months	1,221 (28)	16 (21)	
	> 12 months	2,794 (65)	52 (67)	
Usage of pain medication	no/occasional	1,644 (38)	30 (41)	0.7 (F)
	regular	2,682 (62)	44 (59)	
Employment status	working	1,025 (23)	10 (13)	<0.001 (C)
	unable to work	457 (10)	3 (3.8)	
	unemployed	146 (3.1)	2 (2.5)	
	retired	2,771 (63)	65 (81)	
LSS definition (spondylolisthesis)	Central canal < 3mm	2,224 (48)	2,178 (54)	<0.001 (C)
	Lateral recess < 3mm	919 (20)	660 (16)	
	Central canal > 3mm	1,188 (25)	949 (24)	
	Lateral recess > 3mm	332 (6.9)	224 (5.6)	
Other diagnoses	disc degeneration	61 (1.3)	30 (0.7)	0.01 (F)
	disc herniation	384 (8.2)	313 (7.8)	0.5 (F)
	other	1017 (22)	800 (20)	0.03 (F)

Table S2. Demographic details between the patients who answered and did not answer the PROM questionnaire 1 year postoperatively. The P values are from Fisher's exact test (F), non-parametric bootstrap (B), or a chi-squared test (C) depending on the distribution of the variable.

Variable		Respondent	Non-respondent	P value
Total n, %		3,301 (38)	5,373 (62)	
Age, mean (SD)		67 (10)	68 (10)	<0.001 (B)
Female		1,896 (57)	3,059 (57)	0.7 (F)
BMI (SD)		28.3 (4.7)	28.6 (4.5)	0.03 (B)
Nicotine		324 (13)	343 (17)	<0.001 (F)
Pain duration	< 6 weeks	59 (2.4)	53 (3)	<0.001 (C)
	6– 12 weeks	109 (4.4)	89 (5)	
	3– 12 months	709 (29)	528 (28)	
	> 12 months	1,597 (65)	1,249 (65)	
Usage of pain medication	no/occasional	982 (39)	692 (36)	0.03 (F)
	regular	1505 (61)	1,221 (64)	
Employment status	working	570 (23)	465 (24)	<0.001 (C)
	unable to work	271 (11)	189 (10)	
	unemployed	72 (2.9)	76 (4)	
	retired	1612 (64)	1,224 (63)	
LSS definition (spondylolisthesis)	Central canal < 3mm	1522 (46)	2,880 (54)	<0.001 (C)
	Lateral recess < 3mm	621 (19)	958 (18)	
	Central canal > 3mm	893 (27)	1,244 (23)	
	Lateral recess > 3mm	265 (8.0)	291 (5.4)	
Other diagnoses	disc degeneration	47 (1.4)	44 (0.8)	0.009 (F)
	disc herniation	275 (8.3)	422 (7.9)	0.4 (F)
	other	762 (23)	1055 (20)	<0.001 (F)
Preoperative PROM	ODI, % (SD)	41.8 (16.4)	43.7 (16.6)	
	ODI [CI]	[41.2–42.5]	[42.9–44.4]	<0.001 (B)
	VAS leg pain, % (SD)	59.9 (27.7)	65.6 (25.6)	
	VAS leg pain [CI]	[58.8–61.0]	[64.5–66.8]	<0.001 (B)
	VAS back pain, % (SD)	56.0 (27.9)	61.2 (26.3)	
	VAS back pain [CI]	[54.8–57.1]	[60.0–62.4]	<0.001 (B)

Propensity score matching

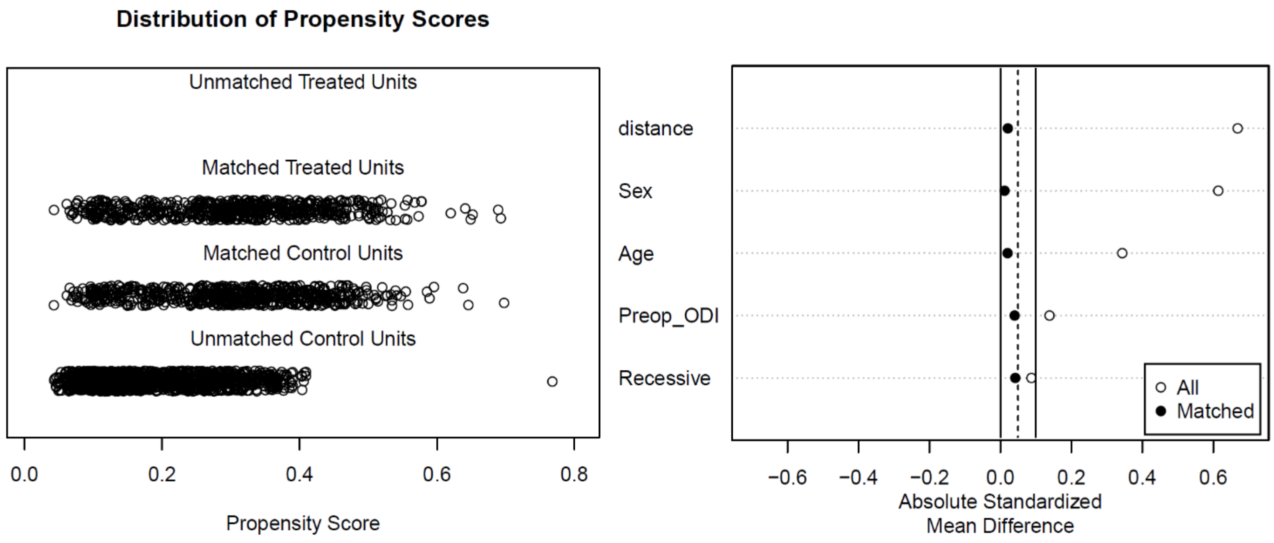

Figure S1. Distribution of propensity scores for matched and unmatched decompression patients (Control) and the distribution for the deco+fusion patients (Treated) [left panel] and the average distance measured by generalized-linear-model-based propensity scores, and the standardized distances for the covariates used in the matching: sex, age, preoperative ODI score, and recessive/central LSS.

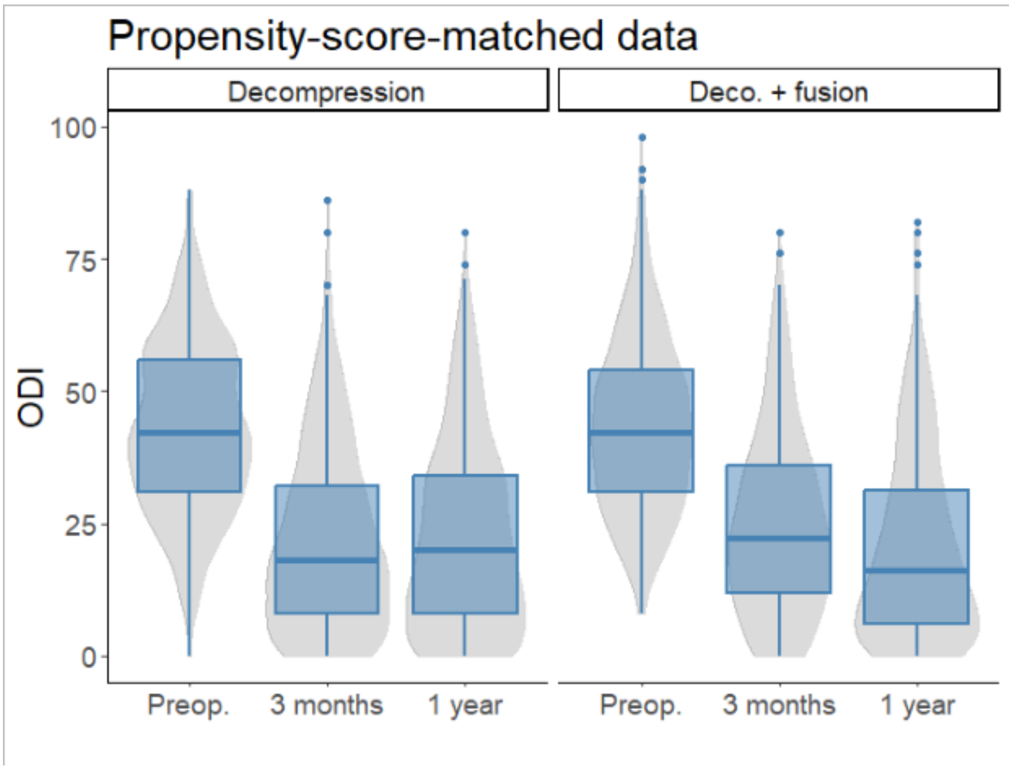

Figure S2. Patient-reported Oswestry Disability Index (ODI) values for lumbar spinal stenosis patients in the FinSpine-registry treated with decompression, and decompression and fusion (Deco. + fusion) for patients matched by propensity score matching.

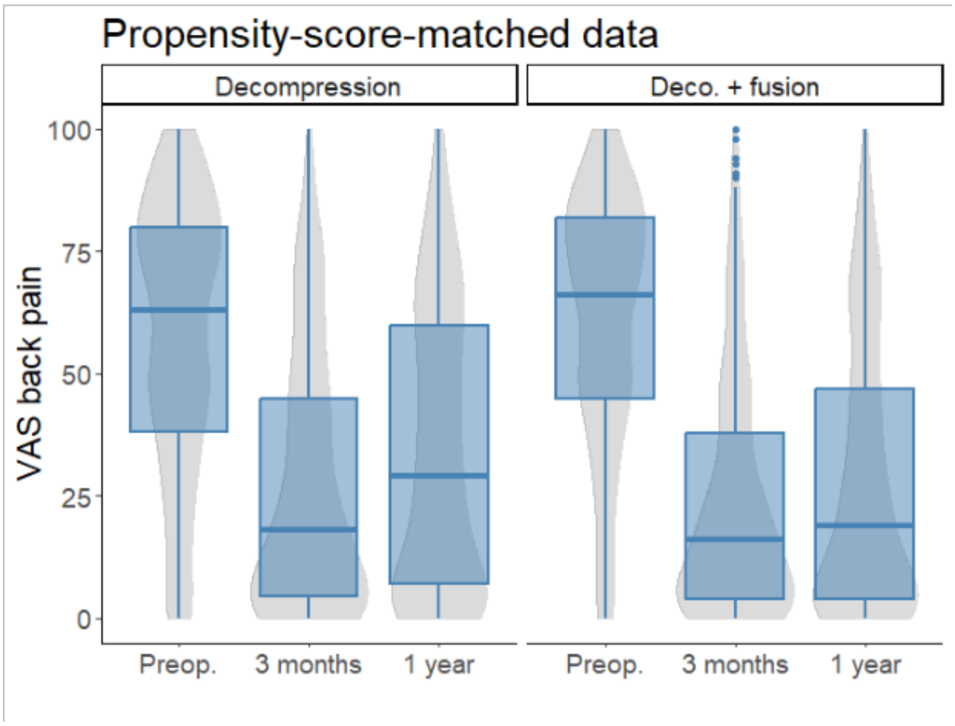

Figure S3. Patient-reported Visual Analogue Scale (VAS) values for lower back pain for lumbar spinal stenosis patients in the FinSpine-registry treated with decompression, and decompression and fusion (Deco. + fusion) for patients matched by propensity score matching.

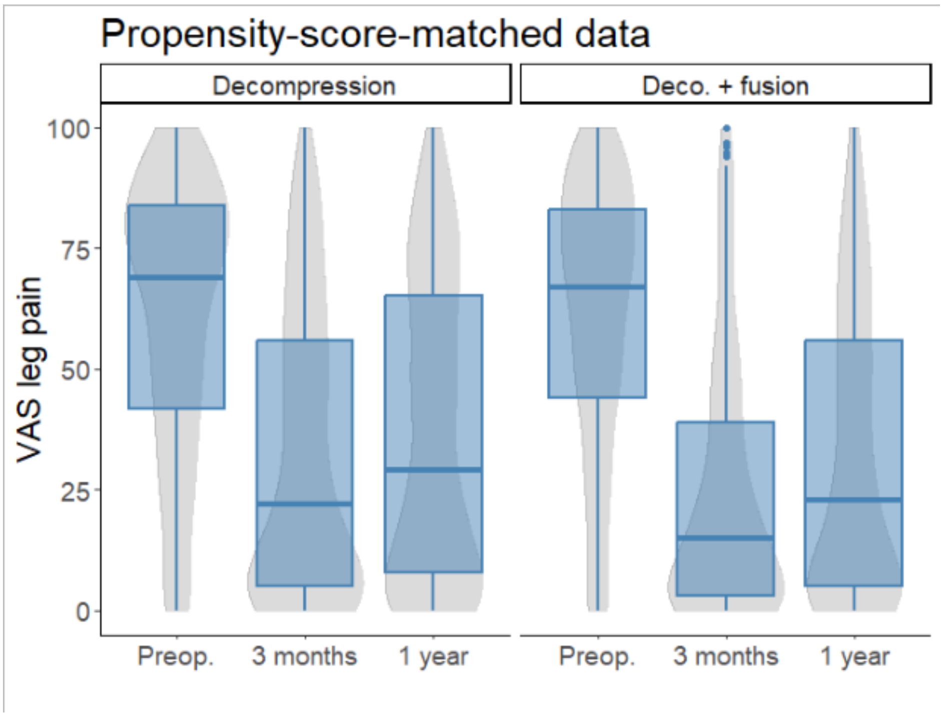

Figure S4. Patient-reported Visual Analogue Scale (VAS) values for leg pain for lumbar spinal stenosis patients in the FinSpine-registry treated with decompression, and decompression and fusion (Deco. + fusion) for patients matched by propensity score matching.

Table S3. Sub-group analysis by stenosis type. Values are mean difference between preoperative and 1 year with (standard deviation) and [95% confidence intervals]

	Central			Recessive		
	Deco.	Deco. + fusion	P-value	Deco. (matched)	Deco.+fusion (full ODI)	P value
ODI	-20.2 (17.9) [-21.9 to -18.6]	-22.5 (18.2) [-24.1 to -20.8]	0.07 (B)	-20.7 (18.4) [-23.6 to -17.9]	-23.9 (26.6) [-26.6 to -21.2]	0.1 (B)
VAS leg pain	-23.5 (36.2) [-27.4 to -19.6]	-29.6 (36.5) [-33.3 to -26.0]	0.02 (B)	-25.9 (35.3) [-32.1 to -19.8]	-27.5 (34.8) [-33.7 to -21.3]	0.7 (B)
VAS back pain	-23.6 (33.5) [-27.0 to -20.2]	-33.2 (34.9) [-36.7 to -29.7]	<0.001 (B)	-26.1 (37.5) [-32.5 to -19.8]	-33.5 (29.4) [-38.8 to -28.3]	0.07 (B)

Table S4. Sub-group analysis for patients with < 3 mm spondylolisthesis. Values are mean difference between preoperative and 1 year with (standard deviation) and [95% confidence intervals]

	Central < 3mm			Recessive < 3mm		
	Deco.	Deco. + fusion	P-value	Deco. (matched)	Deco.+fusion (full ODI)	P-value
ODI	-18.9 (18.1)<br>[-23.0 to -14.8]	-17.0 (20.0)<br>[-21.5 to -12.5]	0.6 (B)	-21.0 (19.5)<br>[-28.3 to -13.8]	-23.1 (18.1)<br>[-29.8 to -16.4]	0.9 (B)
VAS leg pain	-24.9 (35.3)<br>[-33.7 to -16.2]	-29.3 (41.9)<br>[-39.8 to -18.8]	0.5 (B)	-33 (39.8)<br>[-49.7 to -16.4]	-31 (33.9)<br>[-44.8, 17.1]	0.8 (B)
VAS back pain	-24.9 (35.3)<br>[-33.7 to -16.2]	-29.3 (41.9)<br>[-39.8 to -18.8]	0.3 (B)	-17.3 (38.4)<br>[-33.1 to -1.6]	-40.8 (30.8)<br>[-52.7, -29.0]	0.01 (B)

Table S5. Sub-group analysis for patients with ≥3 mm spondylolisthesis.

	Central ≥ 3mm			Recessive ≥ 3mm		
	Deco.	Deco. + fusion	p-value	Deco. (matched)	Deco.+fusion (full ODI)	p-value
ODI	-19.7 (17.1)<br>[-21.7 to -17.8]	-22.8 (18.1)<br>[-24.8 to -20.7]	0.04 (B)	-19.5 (19.8)<br>[-23.6 to -15.3]	-23.7 (15.4)<br>[-26.9 to -20.5]	0.1 (B)
VAS leg pain	-25.4 (35.5)<br>[-30.2 to -20.5]	-29.2 (36.2)<br>[-33.7 to -24.7]	0.2 (B)	-27.6 (35.3)<br>[-36.1 to -19.1]	-26.1 (38.2)<br>[-35.2 to -17.0]	0.8 (B)
VAS back pain	-24.0 (33.2)<br>[-28.4 to -19.7]	-32.3 (34.5)<br>[-36.6 to -28.0]	0.007 (B)	-29.0 (30.5)<br>[-36.0 to -22.0]	-32.3 (28.7)<br>[-39.2 to -25.4]	0.5 (B)

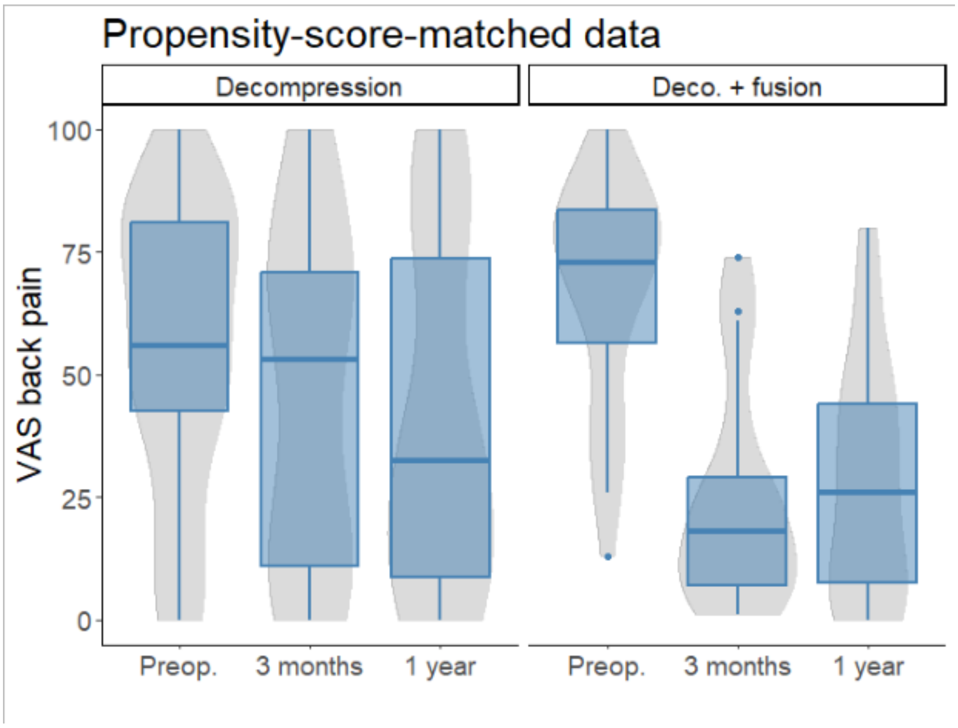

Figure S5. VAS back pain scores for recessive stenosis with <3 mm spondylolisthesis.

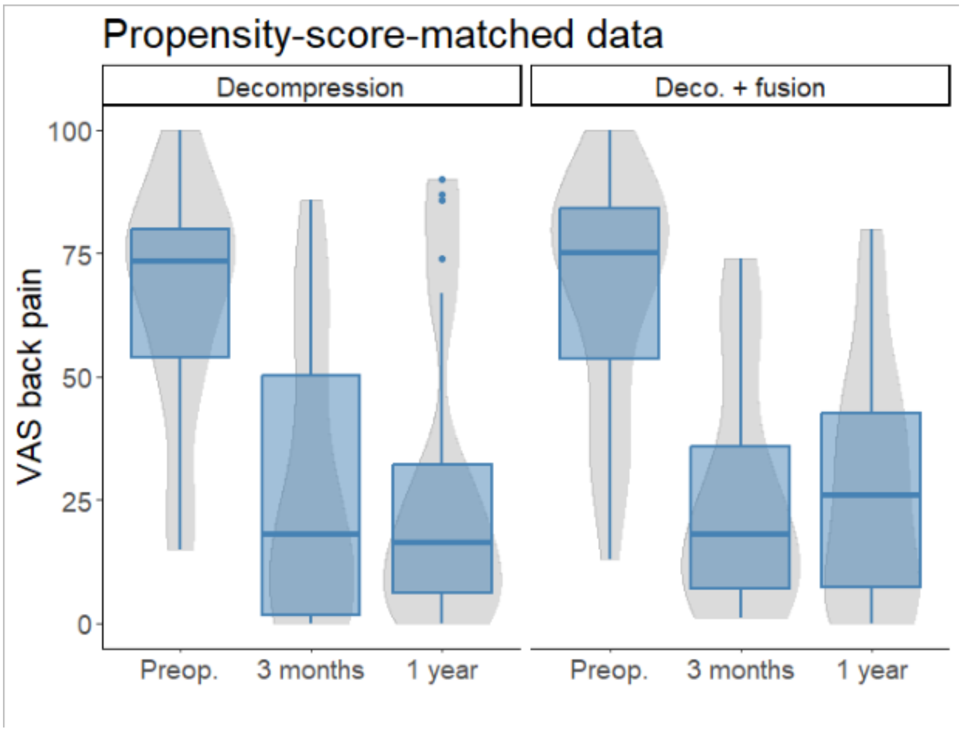

Figure S6. VAS back pain scores for recessive stenosis with < 3 mm spondylolisthesis when propensity score matching uses preoperative VAS back pain score instead of preoperative ODI.
